# Characterization of Protein and Peptide Binding to Nanogels Formed by Differently Charged Chitosan Derivatives

**DOI:** 10.3390/molecules18077848

**Published:** 2013-07-03

**Authors:** Anastasia Zubareva, Alla Ilyina, Aleksander Prokhorov, Denis Kurek, Mikhail Efremov, Valery Varlamov, Sevda Senel, Pavel Ignatyev, Еlena Svirshchevskaya

**Affiliations:** 1 Shemyakin and Ovchinnikov Institute of Bioorganic Chemistry, RAS, Miklukho-Maklaya, 16/10, Moscow 117997, Russia; E-Mails: sashapro2006@yandex.ru (A.P.); mae@ibch.ru (M.E.); 2 Centre of Bioengineering, RAS, Prospect 60-Letia Oktyabrya 7/1, Moscow 117312, Russia; E-Mails: enzyme@biengi.ac.ru (A.I.); svoboda_rus@mail.ru (D.K.); varlamov@biengi.ac.ru (V.V.); 3 Department of Pharmaceutical Technology, Faculty of Pharmacy, Hacettepe University, Ankara 06100, Turkey; E-Mail: ssenel@hacettepe.edu.tr; 4 AMPHORA Laboratories LLC, 5-ya Magistralnaya 11, Moscow 123007, Russia; E-Mail: ips@amphoralabs.ru

**Keywords:** chitosan nanogels, N-acyl chitosan derivatives, acidic proteins, basic proteins, ionotropic gelation, electrostatic interactions

## Abstract

Chitosan (Chi) is a natural biodegradable cationic polymer with remarkable potency as a vehicle for drug or vaccine delivery. Chi possesses multiple groups, which can be used both for Chi derivatization and for particle formation. The aim of this work was to produce stable nanosized range Chi gels (nanogels, NGs) with different charge and to study the driving forces of complex formation between Chi NGs and proteins or peptides. Positively charged NGs of 150 nm in diameter were prepared from hexanoyl chitosan (HC) by the ionotropic gelation method while negatively charged NGs of 190 nm were obtained from succinoyl Chi (SC) by a Ca^2+^ coacervation approach. NGs were loaded with a panel of proteins or peptides with different weights and charges. We show that NGs preferentially formed complexes with oppositely charged molecules, especially peptides, as was demonstrated by gel-electrophoresis, confocal microscopy and HPLC. Complex formation was accompanied by a change in zeta-potential and decrease in size. We concluded that complex formation between Chi NGs and peptide/proteins is mediated mostly by electrostatic interactions.

## 1. Introduction

Chitosan (Chi) is a biocompatible and biodegradable polycationic polymer possessing multiple active groups which can be used to obtain Chi derivatives with variable zeta-potential, solubility, and hydrophobic properties. Chi and its derivatives are widely used to develop nanogels (ChiNGs) as delivery systems for antitumor drugs [1,2,3] or proteins/peptides for various biomedical applications [4,5,6]. ChiNGs for antitumor drug delivery are designed for intravenous injections; and thus they interact with plasma proteins. Plasma protein binding to various nanoparticles is well demonstrated and is thought to depend on electrostatic, hydrophobic, Van Der Waals forces and hydrogen bonds formation with Chi side chains [7]. Primary sorption of proteins is predetermined by proteins present in excess in plasma such as albumin and immunoglobulins. However, in a steady-state condition rarer proteins with higher affinity to polymers such as apolipoprotein E can replace more abundant proteins due to the so called “Vroman effect” [8]. Leading forces determining complex formation between plasma proteins and Chi/ChiNGs are not well established. Most studies are conducted with bovine serum albumin (BSA) showing its high affinity for positively charged Chi derivatives [9,10,11]. Almost no data are published on protein/plasma protein complex formation with negatively charged Chi derivatives such as N-succinoyl chitosan. The only work where negatively charged N-succinoyl chitosan formed complexes with BSA explained it by H-bonds formation and hydrophobic interactions [12]. Contrary to this, oppositely charged polyelectrolytes were effectively used to prepare chitosan films using layer-by-layer adsorption [13]. Sorption by positively charged Chi of positively charged proteins was much less efficient as it was shown for bovine hemoglobin, superoxide dismutase, and insulin [4,9,13,14,15]. 

Chi NGs also are considered as delivery systems for various peptides. Sorption of neutral 10 kDa anti-neuroexcitation peptide by N-trimethylchitosan chloride nanoparticles, determined by bicinchoninic acid assay, was unexpectedly high (80%) [16]. The same high sorption efficacy to glycol chitosan NGs was found for another neutral antiangiogenic small peptide Arg-Gly-Asp [17,18] while binding of glutathione GSH peptide by Chi and Chi/cyclodextrin nanogels was low (7%–20%) [6].

In a recent work of Zou *et al.* [19] combined MALDI-TOF-MS and nano-LC-ESI-MS/MS methods were used to identify plasma proteome of multifunctional chitosan-GMA-IDA-Cu(II) nanospheres. More than 800 peptides and 1,700 unredundant proteins were identified, including relatively low-level proteins (pg/mL of interleukin-3) co-distributed with high-abundance proteins (35–55 mg/mL of serum albumin). In face of high variability in Chi derivatives and huge blood serum proteome, leading forces affecting protein/Chi complex formation must be better characterized. The aim of this work was to verify the hypothesis that electrostatic interactions is a driving force for the formation of complexes between proteins/peptides and chitosan nanogels. 

## 2. Results and Discussion

### 2.1. Production and Characterization of Chitosan NGs Formed by Chitosan-N-acyl Derivatives

**Figure 1:**
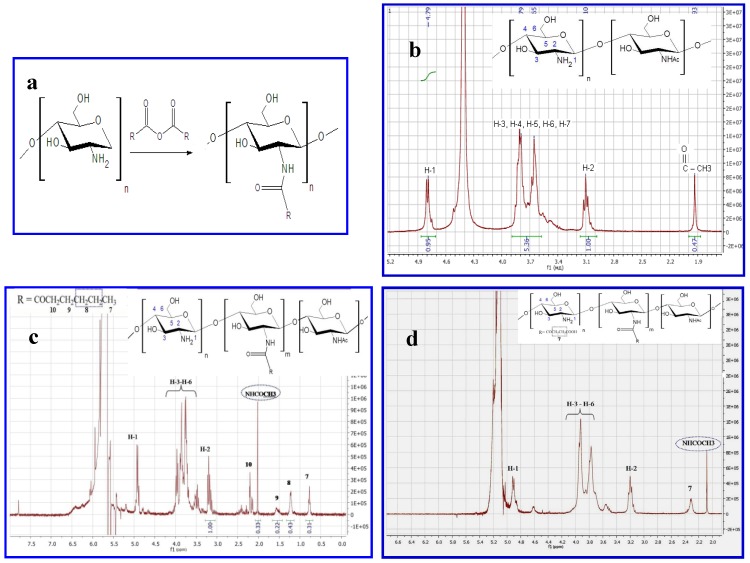
Characteristics of chitosans. (**a**) Reaction between chitosan and carboxylic acid anhydride, where R=(CH2)4CH3- hexanoyl-chitosan; R=(CH2)2 COOH- succinoyl-chitosan. (**b**–**d**) ^1^H-NMR spectra of (**b**) Chitosan with MM 200 kDa and DD 86% (**c**) Succinoyl chitosan with degree substitution (DS) 80% (**d**) Hexanoyl-chitosan with (DS) 10%.

Chitosan derivatives were synthesized by the reaction of acylation with anhydride of carboxylic acid (Figure 1a) and characterized by ^1^H-NMR. Representative ^1^H-NMR spectra of chitosan and N-acyl derivatives are shown in Figure 1b–d. The spectrum of unmodified chitosan is typical and has two characteristic signals (at 3.2 ppm for H-2 protons of glucosamine unit and 1.98 ppm for acetyl groups of N-acetylglucosamine unit). The appearance of the peak at 0.52–2.5 ppm corresponds to the proton of the acyl-group. Substitution degrees were 10% and 80% for hexanoyl-chitosan and succinoyl-chitosan accordingly. 

**Figure 2:**
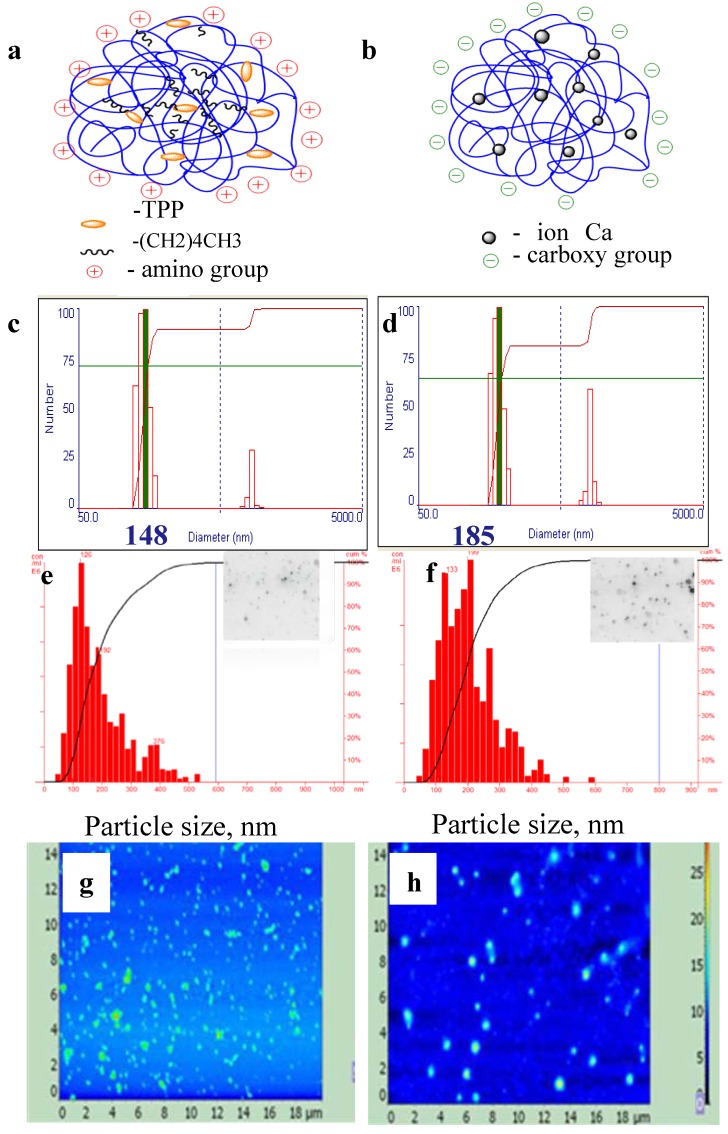
Schematic structures (**a**,**b**), dynamic light scattering (**c**,**d**), tracking analysis (**e**,**f**), and atomic force microscopy (**g**–**i**) of particles from hexanoyl-chitosan formed by tri-poly-phosphate linking (**a**,**c**,**e**,**g**) or from succinoyl-chitosan prepared by Ca^2+^ coacervation method (**b**,**d**,**f**,**h**). Data on NTA are shown as histograms of inserts.

Hexanoyl-chitosan nanogels (HCNGs) were prepared by the ionotropic gelation method; the schematic structure of HCNGs is shown in Figure 2a. Succinoyl-chitosan nanogels (SCNGs) were formed by the Ca^2+^ coacervation method. Their schematic structure is shown in Figure 2b. Earlier HCNGs were prepared and studied by Desai and Park [20], where hexanoyl chitosan was synthesized and nanoparticles were prepared by the same methods. SCNGs prepared by coacervation method were developed by our group previously [21]. Other types of SC nanoparticles are widely described for biomedical applications [22,23,24]. 

The particle size and poly-dispersity were analyzed by dynamic light scattering (DLS); atomic force microscopy; and nanoparticles tracking analysis (NTA) (Figure 2). Analysis of chitosan particles by DLS in multimode demonstrated the presence of two fractions with peaks of 150 and 650 nm for HCNGs (Figure 2c); 180 and 780 nm for SCNGs (Figure 2d). Close results were obtained by DLS by different groups [20,21]. However the process of particle formation is linear and depends on chitosan derivative and counteragent concentrations. Newly formed particles can aggregate however the aggregates may contain various numbers of nanoparticles, otherwise there must be some mechanisms regulating DLS bi-modal distribution. Atomic force microscopy and especially NTA demonstrated that aggregates were distributed randomly with rare particles larger than 500 nm in diameter (Figure 2e–h). Desai and Park [20] have also demonstrated that the diameter of HC nanoparticles was between 54 to 724 nm with a mean diameter of 324 nm.

The difference obtained by DLS and NTA is a result of different approaches in estimating the size of individual particles. DLS measures autocorrelation function and estimates diffusion coefficients for the groups of particles while NTA registers diffusion coefficients for each particle giving a more realistic distribution of particles in poly-disperse cultures. In NTA the rate of particle movement is related to a sphere equivalent hydrodynamic radius as calculated through the Stokes–Einstein equation. The technique calculates particle size on a particle-by particle basis, overcoming inherent weaknesses in ensemble techniques such as dynamic light scattering [25]. Our data correspond to the results obtained, for example, by Mazzarino *et al.*, who measured polycaprolactone particles covered by chitosan [26]. DLS also provided bi-modal distribution while NTA demonstrated a single peak with narrow distribution. 

### 2.2. Formation of Complexes between Chitosan NGs and Differently Charged Peptides

Complexes were formed by passive sorption of differently charged free peptides (Table 1) with either HCNGs or SCNGs. Complexes were thoroughly washed from free peptides and analyzed by HPLC. All experiments were conducted at physiological pH 7.0. Significant sorption was found only between NGs and peptides with opposite charges (Table 1). The highest binding efficiency was observed between peptides P1, P2 with average charge −4 and −3 and positively charged HCNGs. P7 and P8 bearing charge +3 bound negatively charged SCNGs. 

**Table 1:** Efficacy of peptide sorption (%) by nanogels formed by succinoyl- (SCNGs) and hexanoyl- (HCNGs) chitosans.

Peptide	Sequences *	Charge	Sorption, %	
			HCNGs	SCNGs
P1	GWV**D**HFA**D**GY**DE**VIA **	−4	3.8 ± 0.8	0
P2	M**E**LPSFGVSGVN**E**SA**D**M	−3	3.7 ± 0.6	0
P3	**D**QGH**D**TGSASAPAST	−2	1.4 ± 0.2	0
P4	VGI**D**QPPFGIFV	−1	0.8 ± 0.1	0
P5	PSNCHTH**E**GGQLHCT	−1	0	0
P6	C**RE**G**ED**NS**KR**N	0	0	0
P7	**R**MQFSSLTVNV**R**GSGM**R**	+3	0	3.8 ± 0.9
P8	**K**HGTFGPVHFRNQV**K**I**R**	+3	0	7.0 ± 1.1

* Peptides originate from Asp f 2 and Asp f 3 proteins and were used only as examples of peptides bearing different charges. ** Charged amino acids are shown in bold and positively charged ones are also underlined.

Development of nanocarriers for peptides and proteins is in a significant biomedical demand [27,28]. For many purposes peptides are conjugated to core particles. In the work of Kim *et al*. [29], the authors conjugated brain targeting peptide from rabies virus glycoprotein, RVG, to Pluronic-based chitosan nanocarrier. Another example of this approach was used to obtain neutral LTVSPWY peptide conjugated to PEGylated chitosan magnetic nanoparticles for tumor treatment [30]. Conjugation of peptides can be used when the peptides serve only targeting purposes, while in many cases peptides are used as therapies, like leucine(5)-enkephalin, insulin and others [4,6,31]. Thus, delivery systems permitting the release of free bioactive peptides after intracellular degradation of a carrier such as chitosan are highly desired. 

### 2.3. Formation of Complexes between Chitosan NGs and Differently Charged Proteins

Complexes between chitosan NGs and differently charged proteins (Table 2) were formed as above. Proteins were chosen on random to obtain a panel of proteins with different charges. 

**Table 2:** Characteristics of proteins.

Proteins ^a^	Pi ^b^	Charge ^c^	ζ ^d^, [mV]	MM ^e^, [kDa]	Hydrophobicity ^f^
BSA	4.7	−14	−28	66	30
Af2	5.3	−16	−28	37	33
Ins	5.4	−4	−9	5	39
SWM	7.6	+2	+5	17	37
Lys	> 11	+14	+12	14	31

^a^ Proteins used: BSA, bovine serum albumin; Af2, Asp f 2 from *Aspergillus fumigatus*; Ins, human insulin; SWM, sperm whale myoglobin; Lys, chicken lysozyme. ^b^ PI, isoelectric point is taken from the literature. ^c^ Charge is determined by calculating positive and negative amino acids. ^d^ Zeta potential was determined as described in Methods. ^e^ MM, molecular weight, kilodaltons. ^f^ Hydrophobicity is determined by calculation of percentage of hydrophobic acids using Vector NTI® Software (Life Technologies, Grand Island, NY, USA).

**Figure 3:**
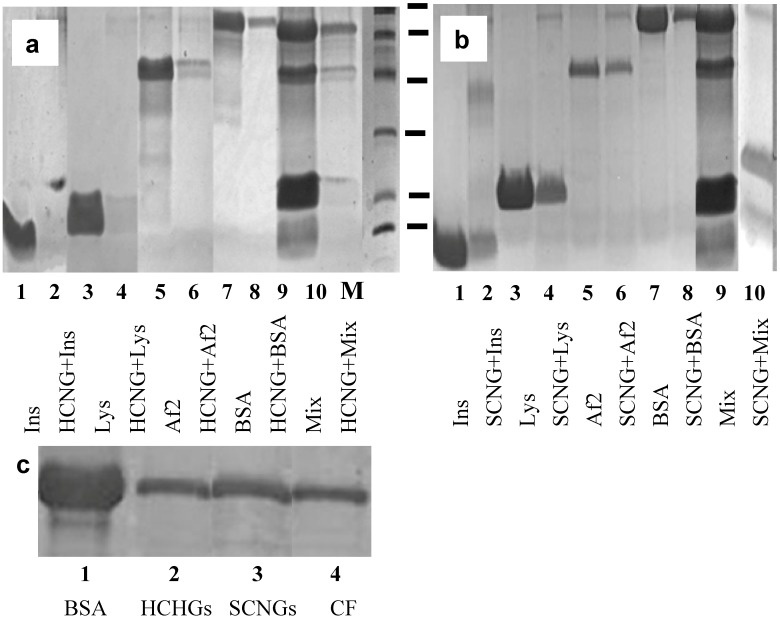
(**a**,**b**). Gel-electrophoresis of loaded proteins: insulin (Ins, 1); lysozyme (Lys, 3); Af2 (5); bovine serum albumin (BSA, 7); mixture of all four proteins (9); and eluted (2, 4, 6, 8, 10 accordingly) from HCNGs (**a**) or SCNGs (**b**). Molecular weight marker (MM) includes 95, 66, 45, 31, 22 and 14 kDa. (**c**). Gel-electrophoresis of BSA loaded on NGs (1), eluted from HCNGs (2); from SCNGs (3) or recovered by centrifugation (CF) without NGs (4).

After incubation with proteins, NGs were separated from unbound proteins by high speed centrifugation and analyzed by gel electrophoresis (Figure 3a,b) while supernatants were used to estimate the amount of unbound proteins by bicinchoninic acid assay **(**BCA assay) (Table 3). The results demonstrated that the highest binding was found between NGs and proteins with different charges. The effect was more pronounced for low molecular weight proteins such as insulin and lysozyme (Figure 3a,b, lanes 1–4) and less evident for the proteins Af2 when analyzed by the electrophoresis (Figure 3a,b, lanes 5–6).

**Table 3:** Sorption (%) of the proteins to nanogels formed by succinoyl- (SCNGs) and hexanoyl- (HCNGs) chitosans.

Protein (ζ, [mV]) pH = 5.5–6.0	ζ, [mV]		Sorption, %	
	HCNGs	SCNGs	HCNGs	SCNGs
Bi-distilled water (0 ± 2)	+27 ± 5	−27 ± 5		
BSA (−15 ± 5)	+14 ± 2 *	−23 ± 5	46 ± 5	47 ± 8 **
Af2 (−16 ± 5)	+15 ± 2 *	−21 ± 2	23 ± 3	0 ± 3
Ins (−9 ± 3)	+9 ± 3 *	−21 ± 3	42 ± 7	0 ± 3
SWM (+5 ± 4)	+26 ± 5	−16 ± 3 *	10 ± 2	36 ± 5
Lys (+12 ± 2)	+26 ± 5	0 ± 2 *	6 ± 3	43 ± 2

* Statistically significant difference between free and loaded NGs (*p* < 0.01). ** BSA sediments from the solution at high speed centrifugation leading to artifact in SCNG complex formation.

When the amount of bound Af2 was estimated by BCA in supernatants, no significant binding was found (Table 3). We obtained unexpected results with bovine serum albumin (BSA): binding of BSA to HCNGs and SCNGs was identified by both gel electrophoresis and BCA (Figure 3a,b, lanes 7–8, Table 3). In an attempt to understand why albumin can bind both negatively and positively charged NGs, we run several experiments and identified the artifact connected to the internal property of BSA to sediment at the centrifugation regime used to separate particles from the free protein (Figure 3c). 

To support the results obtained with individual proteins, all four of them were mixed and loaded onto HCNGs or SCNGs. In the case of mixed proteins HCNGs bound exclusively negatively charged proteins bovine albumin and *Aspergillus* protein Af2 while SCNGs bound insulin and lysozyme both of which were positively charged (Figure 3a,b, lanes 9–10). Some amount of BSA in the sediment from SCNGs could be the result of co-sedimentation during high speed centrifugation. 

Co-sedimentation during centrifugation was found for all proteins used. To decrease this effect, protein solutions were centrifuged at 14,000 rpm for 20 min before the experiments. Pre-centrifugation almost removed co-sedimentation with NGs for all proteins used but not for BSA. 

**Figure 4:**
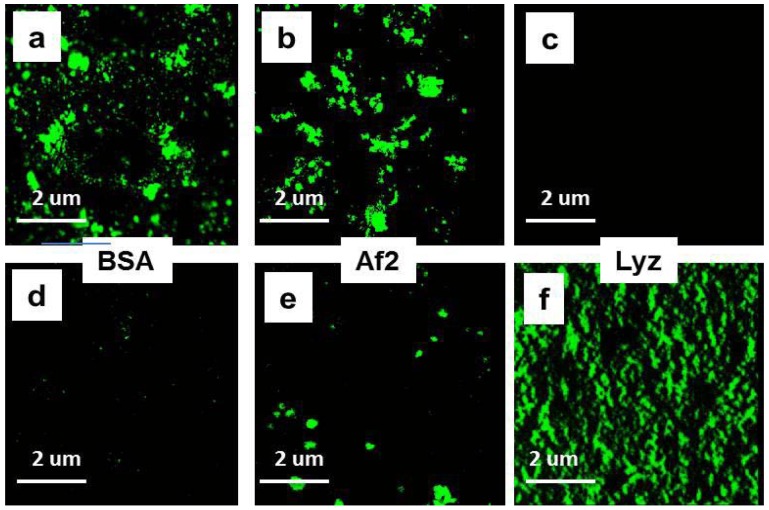
Complexes of HCNGs (**a**–**d**) or SCNGs (**e**–**h**) with FITC-labeled proteins BSA (**a**,**e**), Af2 (**b**,**f**), and Lyz (**c**,**g**) or free FITC (**d**,**h**) visualized by confocal microscopy. Magnification × 3,000.

Thus, another approach was needed to estimate binding of BSA to NGs. The NGs-protein complexes can be distinguished from free protein in the mixture using visualization of loaded NGs by confocal microscopy. To this end proteins were conjugated to FITC and used for complex formation. The results of confocal microscopy demonstrated that positively charged HCNGs predominantly bound negatively charged proteins (Figure 4d–g) and *vice versa*, negatively charged SCNGs bound positively charged proteins (Figure 4h–k). In this case no BSA-SCNGs complexes were registered. To exclude artifacts related to free FITC binding to NGs we also incubated NGs during 30 min with FITC in concentration, equivalent to the amount bound to proteins. No binding was found with SCNG (Figure 4h) and very little binding was found with HCNGs (Figure 4d).

Average sorption for BSA-HCNGs was around 46% which is in a full agreement with the work of Desai and Park [20] where sorption of BSA by HCNGs was around 30%. Formation of complexes between positively charged chitosan derivatives and BSA are widely reported [9,10,11], while systems between negatively charged chitosan derivatives and negatively charged proteins in several papers are found to be formed by unspecific binding [32]. The paper of Rekha and Sharma [21] demonstrated complex formation between laurylsuccinoyl chitosan (negative charge) with insulin (also negatively charged). On average data on complex formation between proteins and negatively charged nanoparticles are scarce. 

It was of interest to determine whether complex formation with chitosan NGs leads to the change in physicochemical parameters of NGs. Estimation of **ζ**-potential was conducted after washing of NGs from unbound proteins. The results demonstrated that **ζ**-potential changes only in the case of complex formation between NGs and proteins (Table 3). For example, decrease in **ζ**-potential was found for HCNGs complexes with BSA, Af2 and Ins, but not for complexes with SWM and Lys (Table 3). 

**Figure 5:**
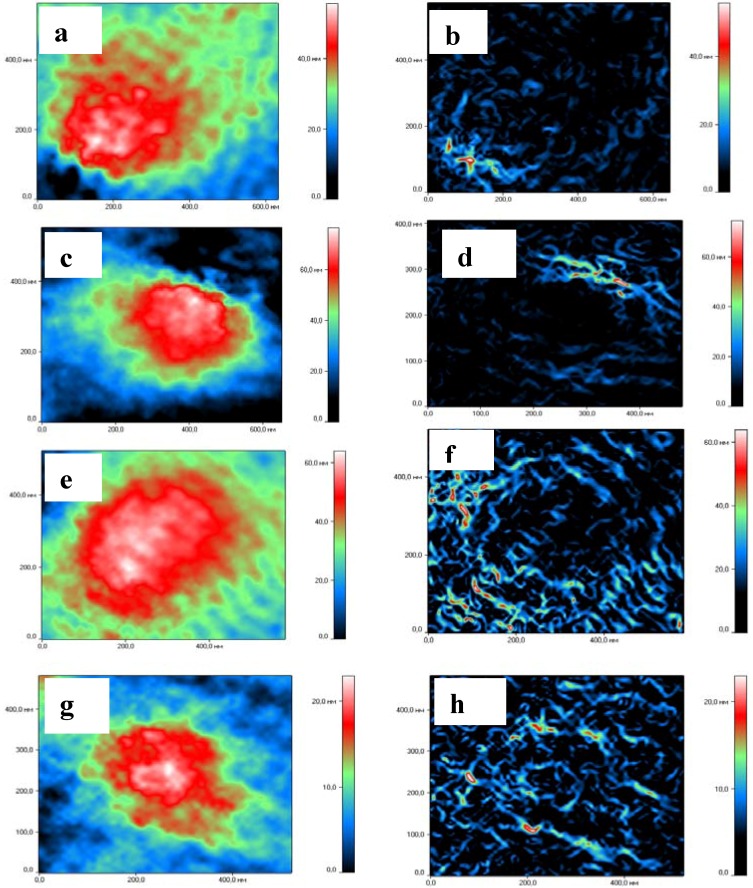
Laser interference microscopy images and size distribution of HCNGs and SCNGs) before and after loading with proteins. HCNGs before (**a**–**b**) and after (**c**–**d**) loading with BSA. SCNGs before (**e**–**f**) and after (**g**–**k**) loading with Lys. External (**a**,**c**,**e**,**g**) and internal (**b**,**d**,**f**,**h**) structures are shown.

The estimation of a diameter of NGs by DLS demonstrated significant decrease in NGs sizes (data not shown). To verify this we used a new technique of laser interfering microscopy permitting not only determination of NG sizes but also visualization of external and internal structures of NGs (Figure 5). In all cases of complex formation, NGs significantly decreased in diameter. For HCNGs with BSA it decreased from 347 nm to 248 nm; for SCNGs – from 326 to 253 nm. The difference between NGs diameters before and after loading with proteins was significant (* *p* < 0.01). The size of complexes between HCNG and Lys; SCNGs and BSA did not change (data not shown). These data supported the results obtained by DLS. 

Contrary to our results, analysis of the literature demonstrated that in some cases diameters of chitosan nanoparticles increased after complex formation with siRNA or peptides [33,34].

HCNGs were spherical in shape (Figure 5a,c), while SCNGs had slightly oval form (Figure 5e,g) both before and after complex formation showing that the structure of NGs is rather rigid. It is difficult to say whether it depends on types of chitosan derivatives or methods of preparation used. NGs formed by different methods had also different internal structures: HCNGs were hollow inside (Figure 5b,e) while SCNGs were filled inside (Figure 5h,k). 

### 2.4. Interaction of Chitosan NGs with Plasma Proteins

**Figure 6:**
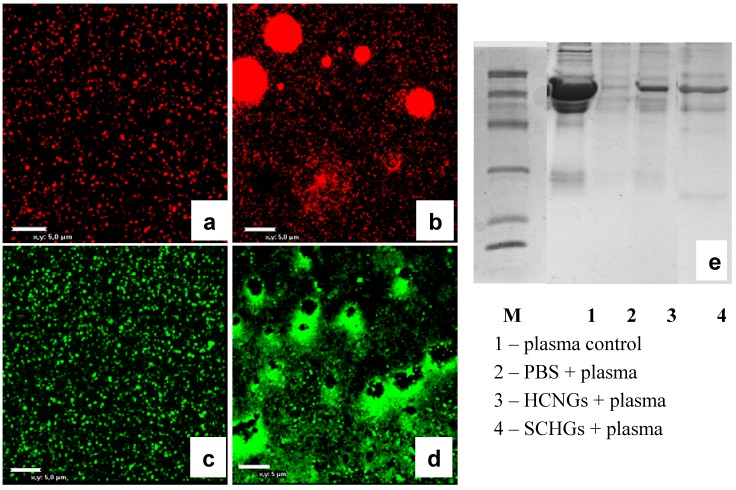
Confocal images of fluorescent HCNGs (**a**,**b**) and SCNGs (**c**,**d**) before (**a**,**c**) and after incubation for 30 min with diluted blood plasma. (**e**) Gel-electrophoresis of blood plasma loaded onto NGs (line 1); collected in the sediment after centrifugation (line 2); or eluted from NGs (lines 3–4) shows differences in protein spectra. Bar corresponds to 5 um.

Finally it was interesting to visualize interaction of chitosan NGs with plasma proteins. To this end we prepared NGs labeled either with red or green fluorescent dyes using a commercial kit, and incubated them with diluted blood plasma for 30 min. Both HCNGs and SCNGs aggregated in plasma demonstrating interaction with plasma proteins (Figure 6a–d) while the pattern of aggregation was different. Positively charged HCNGs evidently aggregated as spheroids (Figure 6b), while negatively charged SCNGs formed a crown around some dark zone (Figure 6d). The reason for this difference is not known. 

NGs after incubation with plasma were washed once in water and absorbed proteins were analyzed by gel-elecrophoresis. NGs with different charge bound different sets of proteins (Figure 6e, lines 3–4). Albumin was found not only in both samples due to above mentioned effect of unspecific sedimentation during centrifugation, but also after centrifugation of plasma without loading onto NGs (Figure 6e, lane 2). 

Chitosan is a very perspective biopolymer due to its safety and versatility. Chi and its derivatives are widely used in experiments to produce nanosized particles as delivery systems for biomolecules such as DNA, proteins, and drugs. In many cases poor understanding of forces responsible for the formation of complexes between Chi NGs and biomolecules of interest makes the investigators prepare conjugates instead of complexes doing the production of particles more time-, money and labor-consuming. Preparation of stable complexes between Chi NGs and bio-molecules instead of conjugates also preserves bioactivity of the cargo which is especially important for small molecules such as peptides and drugs. Here we demonstrated that complex formation between Chi NGs and peptides/proteins is mainly determined by electrostatic interactions. However, other mechanisms such as hydrophobic, Van Der Waals forces and hydrogen bonds formation are also operative. For example, the binding by HCNGs of insulin, a protein with MW 5 kDa and zeta-potential −8 mV, was higher than the binding of Asp f 2, 37 kDa protein with −28 mV zeta-potential (Table 3). The same dependence was found for lysozyme and myoglobin bound to SCNGs: larger protein SWM formed complexes less efficiently (although the potential was also lower) than smaller Lys. We hypothesized that primary interaction of proteins with Chi NGs is determined by electrostatic interaction and then smaller proteins are entrapped inside the particles while larger ones are only partially immersed into the NGs and dissociate with time. On the other side peptides were entrapped less efficiently (maximum for P8 was 7%, compare with 42% for insulin) than even small proteins (Table 2, Table 3). In other words it is likely that there is an optimal protein mass/length to be entrapped within Chi NGs. For the first time we used laser interference microscopy to visualize the NG structures. As shown in Figure 5b,h chitosan NGs are not homogenous and have either hollow (HCNGs) or solid (SCNGs) cores and a shell which serves as a primary contact interface. When NGs interact with proteins with opposite charge both NGs and charged proteins behave as polyelectrolytes and form a dense interface which keeps proteins bound to the surface of NGs. Formation of polyelectrolyte zone between chitosan and protein leads to the decrease in the size of NGs. 

Finally we analyzed the effect of blood plasma proteins on NGs behavior and found out a severe aggregation induced by various inter-particle interactions. The aggregation was found both for HCNGs and SCNGs, however the forms of aggregates differed between NGs. The aggregates could be easily disrupted by pipetting showing the weak forces keeping NGs in associates. Formation of aggregates is a result of residual charge of the nanogels. Serum albumin normally aggregates with many blood proteins protecting them during their transport via blood to their targets. As albumin is a self protein there is no harm in the formation of additional shell over the nanogels. 

## 3. Experimental

### 3.1. Materials

Proteins bovine serum albumin (BSA), sperm whale myoglobin (SWM), lysozyme (Lyz) (all from Sigma*-*Aldrich Chemie Gmbh Munich, Germany), human insulin (Ins) (IBCh, Moscow, Russian Federation) were used in the study. Asp f 2 from *Aspergillus fumigatus* was a gift from Prof. V.P. Kurup (Medical College of Wisconsin, Milwaukee, WI, USA). Chitosan with molecular weight (MW) 200 kDa and deacetylation degree (DD) 86% and succinoyl chitosan with MW 340 kDa and substitution degree 80% were purchased from ZAO «Bioprogress» (Moscow region, Russian Federation). Chitosan and succinoyl chitosan were precipitated by the addition of 12 wt% aqueous ammonium solution or 30% CH_3_COOH (Reachim, Moscow, Russian Federation) accordingly. Precipitates were collected and vacuum dried. Molecular weight of chitosan after purification was 200 kDa as determined by viscosimetry. Hexanoyl anhydride and fluorescein isothiocyanate (FITC) were obtained from Fluka (Sigma*-*Aldrich, Munich, Germany). N-hexanoylchitosan (HC) derivative was obtained using hexanoic acid anhydride as described in [35]. The yields of N-acylchitosan derivative after dialysis and freeze-drying cycles ranged from 80% to 85%. Degrees of N-substitutions (DS) were determined by ^1^H-NMR (Figure 1). ^1^H-NMR spectra were measured in deuterium chloride (DCl) for HC and D_2_O for SC using a 400 MHz spectrometer Bruker AMX (Vernon Hills, IL, USA).

### 3.2. Preparations of Chitosan Nanogels

#### 3.2.1. N-hexanoylchitosan Nanogels (HCNGs)

HCNGs were formed by the ionotropic gelation method [36]. For this 0.25% (w/v) HC solutions were prepared in 0.4% acetic acid (Reachim, Moscow, Russian Federation). Tripolyphosphate (TPP) solution (Sigma*-*Aldrich, Munich, Germany) (1.0%) was added dropwise under magnetic stirring at 30 rpm until opalescence occurred, which was estimated by a Specol 11 spectrophotometer (Carl Zeiss Jena, Germany) at 590 nm. The resulting HC particle suspension was subsequently centrifuged at 14,000 rpm for 20 min. The precipitated particles were re-suspended in bi-distilled water. The yield of particles ranged from 8 to 10%. Concentrations of HC and SC were selected as giving the highest yield NGs.

#### 3.2.2. Succinoylchitosan Nanogels (SCNGs)

SCNGs were formed by the coacervation method as previously published [21,36]. Briefly, 1% CaCl_2_ (Sigma) was added dropwise to 0.15% SC pre-diluted in bi-distilled water under constant stirring for 20 min at 30 rpm and 22 °C. SCNGs were separated by centrifugation (14,000 rpm, 20 min) and then re-suspended in bi-distilled water. Aliquots of the suspension were freeze dried to estimate the amount of SC inserted in the nanostructures. The yield of particles was about 8%. 

### 3.3. Characterization of Chitosan Nanogels

#### 3.3.1. Dynamic Light Scattering (DLS)

The mean size and zeta potential of the ChiNGs were determined by 90 Plus Particle Size Analyzer (Brookhaven Instruments Corporation, Vernon Hills, IL, USA). Parameters of nanogels were measured in bi-distilled water at a scattering angle 90° and 661 nm wavelength. The zeta potential of the system was determined in 10 mM KCl (Sigma) using identical equipment with an additional ZetaPALS apparatus. Measurement was performed at 25.0 ± 0.1 °C and then measurements were taken for each sample.

#### 3.3.2. Atomic Force Microscopy (AFM)

Diluted solutions of chitosan NGs in bi-distilled water were dried on a freshly cleaved mica surface and analyzed using NTEGRA Prima equipment (NT-MDT, Moscow, Russian Federation) in a semi-contact mode with NSG01 cantilevers.

#### 3.3.3. Nanoparticle Tracking Analysis (NTA)

NTA experiments were performed using a NanoSight NS500 system (NanoSight, Wiltshire, UK). Video images of particle movement under Brownian motion were analyzed by NTA analytical software version 2.2. The measurements were made at room temperature and 60 s capture of video clips.

#### 3.3.4. Laser Interference Microscopy

ChiNGs visualization was accomplished with a MIM-321 laser interference microscope (AMPHORA Laboratories LLC, Moscow, Russian Federation). The spatial resolution up to 0.1 nm vertical (Z) and 10 nm lateral (XY) was achieved with Olympus MPLFLN 100 × 0.9 dry objective. The 10 μL diluted solution of NGs were placed on the mirror glass and dried. The gradient filtering was applied to visualize the shape and the internal structure of ChiNGs.

### 3.4. Complex Formation between Chitosan Nanogels and Various Peptides

Peptides (Table 1, 1 mg/mL) were mixed with HCNGs (500 µg/mL) or SCNGs (400 µg/mL) in bi-distilled water at w/w ratio 1:2. Different concentrations of NGs used for this experiment resulted from the procedure of NG preparation where the concentration of chitosan derivatives were chosen to give the highest yield of NGs. Complexes were formed during 30’ at room temperature and washed by centrifugation. Peptides bound to NGs were separated by 40 µL 0.3 NaCl (Reachim, Moscow, Russia). Sorption was determined by calibrated HPLC using a Perkin Elmer column (ODS C_18_; 4.6 × 250 mm, 5 µm) in acetonitryl gradient 0–100% in 0.1% trifluoroacetic acid (TFA). Elution was conducted at 1.2 mL/min velocity; registration was fulfilled at 214 nm. Sorption was calculated as the ratios of area under the curves in control and experimental samples. Elution time for free peptides and peptides absorbed onto NGs were equal. 

#### 3.4.1. Complex Formation between Chitosan Nanogels and Various Proteins

HCNGs (500 µg/mL) and SCNGs (400 µg/mL) in bi-distilled water were mixed with protein solutions (BSA, SWM, Lys, Asp f 2, Ins) at w/w 2:1 ratio. The mixtures were incubated at room temperature for 30’, than NGs were separated from unbound proteins by centrifugation at 10,000 rpm for 30 minutes. Supernatants were transferred to 96-well plates and analyzed by BCA method (Promega, Madison, WI, USA). Percentage of sorption was calculated as ratios of amount of proteins identified in supernatants after sorption of proteins by NGs to total amount loaded onto NGs. Proteins bound to NGs were analyzed by 12% SDS-PAGE.

#### 3.4.2. Visualization of NGs

Proteins were labeled with FITC according to the manufacture protocol (Innova Biosciences, Cambridge, UK). Complexes of NGs with FITC-labeled proteins, prepared as described above, were dropped onto poly-l-lysine covered cover slips and incubated for 3 h. Samples were fixed with 1% paraformaldehyde, washed, and polymerized with Mowiol 4.88 medium (Calbiochem, Darmstadt, Germany). Slides were analyzed using Eclipse TE2000 confocal microscope (Nikon Instruments Europe BV, Amsterdam, Netherlands).

#### 3.4.3. Chitosan NG Binding to Blood Plasma Proteins

For visualization of particle aggregation in blood plasma, HCNGs were labeled with FITC and SCNGs—with rhodamine using the kit from Innova Biosciences. Fluorescent NGs were mixed at 1/1 v/v ratio with 1/15 diluted human blood plasma. Samples were loaded onto poly-l-lysine covered cover slips, incubated for 30’ and processed as described above. An aliquot was incubated in 96-well round bottom plate and collected for 12% SDS-PAGE analysis after washing by centrifugation. Particle aggregation in plasma was visualized by confocal microscopy. 

### 3.5. Statistics

Statistical analysis was performed using Student's t-test. Comparison values of *p* < 0.05 were considered statistically significant.

## 4. Conclusions

Chitosan is a biodegradable positively charged polymer with multiple groups able to interact with various molecules via electrostatic, hydrophobic, Van Der Waals forces and hydrogen bond formation. Our data demonstrate that electrostatic forces predetermine the formation of polyelectrolyte complexes between chitosan nanoparticles and peptides/proteins. Formation of the complexes is accompanied by zeta potential compensation and size decrease possibly due to the dense packing of chitosan side chains. These parameters can be used to control the process of complex formation. Percentage of sorption directly depends on the charge however the type of a protein can also play a role. In many applications conjugation of drugs or macromolecules to the carrier should be avoided either to protect biological activity or facilitate cargo release. In this case it is highly recommended to select chitosan derivatives with opposite charge to the substance of interest. 
